# Microgravity Effect on Pancreatic Islets

**DOI:** 10.3390/cells13181588

**Published:** 2024-09-21

**Authors:** Lukas Zeger, Povilas Barasa, Yilin Han, Josefin Hellgren, Itedale Namro Redwan, Myrthe E. Reiche, Gunnar Florin, Gustaf Christoffersson, Elena N. Kozlova

**Affiliations:** 1 Regenerative Neurobiology, Department of Immunology, Genetics and Pathology, Uppsala University, 75108 Uppsala, Sweden; lukas.zeger@igp.uu.se (L.Z.); yilin.han@igp.uu.se (Y.H.); 2 Department of Biological Models, Institute of Biochemistry, Vilnius University, LT-08662 Vilnius, Lithuania; povilas.barasa@gmc.vu.lt; 3 CELLINK Bioprinting AB, Langfilsgatan 7, 41277 Gothenburg, Sweden; josefin.hellgren@cellink.com (J.H.); inr@cellink.com (I.N.R.); 4 Department of Medical Cell Biology, Science for Life Laboratory, Uppsala University, 75310 Uppsala, Sweden; myrthe.reiche@mcb.uu.se (M.E.R.); gustaf.christoffersson@mcb.uu.se (G.C.); 5 Swedish Space Corporation, 75123 Solna, Sweden; gunnar.florin@sscspace.com

**Keywords:** microgravity, 3D printing, proliferation, delayed effect, beta-cell, neural stem cell, diabetes, pancreatic islets

## Abstract

We previously demonstrated that boundary cap neural crest stem cells (BCs) induce the proliferation of beta-cells in vitro, increase survival of pancreatic islets (PIs) in vivo after transplantation, and themselves strongly increase their proliferation capacity after exposure to space conditions. Therefore, we asked if space conditions can induce the proliferation of beta-cells when PIs are alone or together with BCs in free-floating or 3D-printed form. During the MASER 15 sounding rocket experiment, half of the cells were exposed to 6 min of microgravity (µg), whereas another group of cells were kept in 1 g conditions in a centrifuge onboard. The proliferation marker EdU was added to the cells just before the rocket reached µg conditions. The morphological assessment revealed that PIs successfully survived and strongly proliferated, particularly in the free-floating condition, though the fusion of PIs hampered statistical analysis. Proliferation of beta-cells was displayed in 3D-printed islets two weeks after µg exposure, suggesting that the effects of µg may be delayed. Thus, PIs in 3D-printed scaffolds did not fuse, and this preparation is more suitable than free-floating specimens for morphological analysis in µg studies. PIs maintained their increased proliferation capacity for weeks after µg exposure, an effect that may not appear directly, but can emerge after a delay.

## 1. Introduction

Differences in gravitational acceleration experienced by the body during space travel can lead to various pathophysiological conditions. These include adverse effects on the cardiovascular system, alterations in fluid distribution, immune system deterioration, muscle atrophy, osteopenia, and even a simple loss of body mass [1]. Type 1 diabetes, an autoimmune disease, is characterized by the destruction of highly specialized endocrine pancreatic beta-cells resulting in impaired insulin production, ultimately leading to the deterioration of glucose homeostasis in the human body [2,3]. The adult pancreas has limited regenerative potential, and the identification of approaches that promote beta-cell renewal is thus of great importance [4]. It was shown that neural crest stem cells (NCSCs) regulate beta-cell mass during development [5]. We previously determined that the derivates of NCSCs—the boundary cap—have a strong in vitro effect on beta-cell proliferation [6]. Boundary cap neural crest stem cells (BCs) are a transient group of cells located at spinal root exit and entry points during embryonic development [7] and have the potential to differentiate into neurons and glia in vitro [8] and after transplantation into the peripheral [9] or central nervous system [10,11] in vivo. In addition, BCs stimulate the proliferation of co-cultured insulin-producing beta-cells [12] and improve the survival and function of co-transplanted insulin-producing mouse and human pancreatic beta-cells [6,13]. Furthermore, BCs also protect co-cultured beta-cells from cytokine-induced death [14,15] and are resistant to oxidative stress themselves [16]. In addition, BCs co-transplanted with mouse and human pancreatic islets (PIs) increase islet vascularization and innervation, most likely mediated by diffusible factors and matrix modeling enzymes released from the BCs [6,14,15,16,17]. We recently observed the ability of BCs to promote vascular growth in vitro in 3D-printed scaffolds and after implantation in vivo [18], suggesting that BCs might have a beneficial effect on PIs when printed together. We also found that the increased proliferation by BCs after exposure to µg is due to the activation of genes, regulating proliferation and neuronal fate [19]. Therefore, we investigated if space conditions onboard the MASER 15 Suborbital Express could promote the proliferation of beta-cells in PIs and if this effect can be increased when they are cultured together with BCs or through structural support from 3D bioprinting.

## 2. Materials and Methods

### 2.1. Preparation and Culture of Pancreatic Islets (PIs) and Boundary Cap Neural Crest Stem Cells (BCs)

The Regional Ethics Committee for Research on Animals approved all animal procedures. Islets were isolated from the pancreas of adult NMRI mice and cultured free-floating in RPMI 1640 medium (Sigma-Aldrich, Schnelldorf, Germany) supplemented with L-glutamine (2 mmol/L; Sigma-Aldrich, Schnelldorf, Germany), benzylpenicillin (100 U/mL; Roche Diagnostics, Indianapolis, IN, USA), and 10% (*v*/*v*) fetal calf serum (Sigma-Aldrich, Schnelldorf, Germany) as previously described [15]. BC neurospheres were prepared from transgenic mice harboring red fluorescent protein (RFP) under the universal actin promoter as previously described and cultured in proliferation medium (DMEM/F-12 medium (Invitrogen, Gothenburg, Sweden, 31330-038) supplemented with B27 (Invitrogen, Gothenburg, Sweden 17504-044), N2 (Invitrogen, Gothenburg, Sweden, 17502-048) and containing 20 ng/mL bFGF (Invitrogen, Gothenburg, Sweden, 13256-029) and 20 ng/mL EGF (R&D system, Dublin, Ireland, 236-EG)) [13]. BCs were cultured at 0.5 × 10^5^ cells/cm^2^ in 6-well low-adhesion plates (Corning, Duderstadt, Germany, 3471) and trypsinized every other day for two weeks. A total of 30 BC spheres were placed in each membrane tube with proliferation medium (the plastic tube with the lid), and 30 islets alone or as co-cultures (15 BC spheres + 15 islets) were placed in each membrane tube with RPMI medium. The membranes were then assembled into cassettes for MASER 15, and control groups were maintained on the ground. The proliferation marker EdU was added to the cells just prior to µg exposure. Five hours post-flight, the medium was removed, and cells were fixed for morphological assessment.

### 2.2. Three-Dimensional Printing of Pancreatic Islets (PIs) and Boundary Cap Neural Crest Stem Cells (BCs)

BCs and PIs were delivered to CELLINK (www.cellink.com; accessed 18 September 2024) for 3D printing. The bioink CELLINK LAMININK 521 was used for the printed scaffolds. For the 3D-printed scaffolds of BC+PI, we mixed in 1.7 mL bioink with 0.26 mL cell suspension for a final volume of 1.96 mL and a total of 17.25 million BCs as well as 290 islets/mL bioink. For the single culture of BCs, a bioink volume of 1.7 mL was mixed with 0.190 mL cell suspension for a total volume of 1.89 mL for a final cell concentration of BCs of 9.1 million cells/mL bioink. For the PI-alone cultures, a final concentration of 300 PIs/mL of bioink (568 islets in 1.89 mL bioink) was prepared. BCs alone, islets alone, and the co-culture (PI+BC) were mixed with the bioink and printed as droplets using BIO X (CELLINK, Gothenburg, Sweden, #D16110020717) one week prior to launch. Five islets, BCs, or PI+BC droplets were placed in each membrane and installed in the cassettes, as described below.

### 2.3. Preparation and Loading Pancreatic Islets and Boundary Cap Neural Crest Stem Cells into Sounding Rocket

The final preparations prior to launch were performed in the Esrange Space Center biolaboratory (www.sscspace.com; accessed 18 September 2024). The custom-made experimental module BIM, developed within European Space Agency (ESA) programs, was used. SIOUX Technologies (https://www.siouxtechnologies.com; accessed 18 September 2024) provided the special hardware (Supplementary Figures S1–S3) for installing materials in the MASER 15 sounding rocket. This hardware assembled the cassettes, where the membranes with the cells were placed. The center membrane of each triple membrane contained the cells. In contrast, side membranes were designed to transfer their contents into the main compartment through an electronically activated plunger mechanism. Whereas the EdU from one side compartment was delivered at the moment of exposure to µg (1 mL, 20 μM), the other side compartment contained a fixative (4% paraformaldehyde in the final concentration) as a precaution in case the material would not be found within 4 h after landing. After filling all compartments, the membranes were sealed with respective membrane lids. Using needles (G25, G27), the remaining air bubbles within the closed compartments were manually extracted. Then, the metal lids and electronic plunger mechanisms were screwed on the base block. Thus, completing the assembly of the experimental cassette. Lastly, SIOUX performed various control steps in terms of potential leakage, remaining air bubbles, and overall weight of the cassettes. All samples were divided into two groups; one half was placed in a centrifuge installed in the experimental module onboard to keep 1 g conditions, while the other half of cells were subjected to µg.

### 2.4. Post-Flight Processing of Pancreatic Islets (PIs) and Boundary Cap Neural Crest Stem Cells (BCs)

After landing, the specimen module was delivered to the laboratory at Esrange. Specimens that were treated with EdU during the flight were fixed with 4% phosphate-buffered paraformaldehyde 5 h after the landing. Specimens were immersed overnight in phosphate buffer with 10% sucrose, placed in Tissue-Tek^®^, (Sakura Finetek, Alphen aan den Rijn, The Netherlands) cryosectioned at a thickness of 12 µm and processed for the following stainings: EdU Click-iT Plus EdU Alexa Fluor 647 Imaging Kit (Invitrogen, Eugene, ON, USA, C10640, 10 µM); Ki-67 (Invitrogen, Gothenburg, Sweden, AB_2574235); insulin (Fitzgerald, Täby, Sweden, #20-IP30, 1:250); secondary antibody: Alexa Fluor 488-conjugated donkey anti-guinea pig (Fitzgerald, #706-545-148, 1:1000). Labeled sections were analyzed in a Nikon Eclipse E800 epifluorescence microscope and a Zeiss LSM710 confocal laser scanning microscope. The 3D-printed droplets were analyzed for cell viability and cell number one and two weeks after space exposure, using labeling protocols based on staining live cells with Calcein AM (Invitrogen, #15560597, 5 µM) and dead cells with propidium iodide (Sigma-Aldrich, #818451, 5 µM) for islet cell analysis, whereas NucGreen™ Dead 488 ReadyProbes (Invitrogen, #R37109, 1:1000), which stains dead cells, and RFP (for total cell count) were used for BC analysis.

After the spaceflight, the 3D droplets were returned to CELLINK for cell viability and cell number analysis in the different space conditions (1 g and µg) compared to ground control. The 3D-printed droplets were thereafter sent to Uppsala for analysis of cell proliferation. Three days post-flight, all retrieved bioprinted PIs and PI+BC samples were covered in Tissue-Tek and frozen. The embedded samples were cut into 10 µm thick sections using a cryotome, placed on Superfrost glass slides (Thermo Scientific, Breda, The Netherlands), and stored at −20 °C until staining. For immunostaining, EdU Click-iT Plus EdU Alexa Fluor 647 Imaging Kit (Invitrogen, Eugene, OR, USA, C10640, 10 µM), anti-Ki67 (Invitrogen, AB_2574235, 10 µg/mL), and anti-insulin (Fitzgerald, #20-IP30) primary antibodies were used, as well as Hoechst 33342 dye (Invitrogen, 2433875, 1:4000) to stain nuclei. Secondary antibodies were Alexa Fluor 488-conjugated donkey anti-guinea pig (Fitzgerald, #706-545-148, 1:1000). Images were taken using a Zeiss LSM700 confocal microscope to obtain and merge images of three layers per sample.

### 2.5. Image Analysis

Image analysis to detect insulin, Ki67, or EdU labeling was performed using ImageJ as follows: the multi-Z-level images of one PI or PI+BC slice were merged into one by calculating average pixel brightness. For PI+BC samples, the BC fluorescence image was used to create a mask. This mask was then used to remove the BC signal from Hoechst, insulin, Ki67, or EdU fluorescence, effectively leaving only the PI signal. Subsequently, for PI and PI+BC samples with the BC signal masked, the background signal was removed from the images of nuclei using the rolling ball method, and the nuclear zones were cataloged with the Analyze Particles function. Then, the fluorescence of insulin, Ki67, and EdU was measured in these zones. The threshold for insulin, Ki67, or EdU positivity was calculated by averaging the lowest fluorescence intensity nuclear zones and adding ten times the standard deviation (SD) of these lowest intensity zones. Relative insulin detection was calculated by measuring the total fluorescence intensity of the samples and dividing the value by the number of beta-cells so as to normalize the values according to the size of the islet sample [20].

### 2.6. ELISA

Supernatants were analyzed for insulin and proinsulin content using ELISA kits (Mercodia, Uppsala, Sweden, #10-1247-01 and #10-1232-01). Colorimetric detection was performed using a plate reader (Tecan Spark) (Tecan, Männedorf, Switzerland).

### 2.7. Statistical Analysis

One-way ANOVA + Tukey HSD post hoc tests were performed to determine possible differences between the conditions; repeated-measure ANOVA and Bonferroni Corrections were used for paired measurements over time.

## 3. Results

### 3.1. Viability of Boundary Cap Neural Crest Stem Cells (BCs)

**Figure 1:**
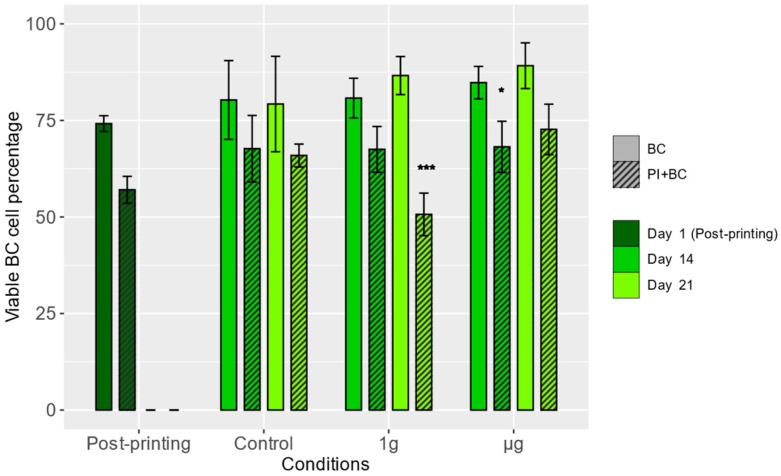
BC viability in bioprinted scaffolds. The viability percentage was calculated by dividing the number of live BCs by the total number of BCs observed in images of the samples. Means (columns) and standard deviations (error bars) are displayed. Stars denote statistically significant (*—*p* < 0.05, ***—*p* < 0.001) differences between the means measured in BC and PI+BC samples on a specific day and treated with specific conditions. *N* = 3 (post-printing groups), *N* = 4 (other groups).

Using fluorescent probes specific for dead cells and the BC-specific RFP fluorescence, the numbers of live and dead BCs in images of the samples were evaluated immediately after bioprinting and one and two weeks after the flight (14 and 21 days after bioprinting) (Figure 1).

Immediately after bioprinting, the BC viability was determined to be 74.17 ± 2.05% for the BC-alone group and 57.04 ± 3.48% for the PI+BC group. In general, BCs in the co-culture samples exhibited a lower level of viability, with statistically significant differences observed in 1 g condition samples two weeks after spaceflight and µg condition samples one week after spaceflight. There were no statistically significant differences between any of the Day 1 experimental groups. It shows that in µg condition, regardless of whether BCs are alone or together with PIs, they have similar viability two weeks after landing, whereas in 1 g conditions, the viability of BCs cultured together with PIs was significantly reduced. This shows that µg might have a positive effect on BC survival with the delay and can bring them back to normal conditions, similar to ground control.

### 3.2. Proliferation of Boundary Cap Neural Crest Stem Cells (BCs)

**Figure 2:**
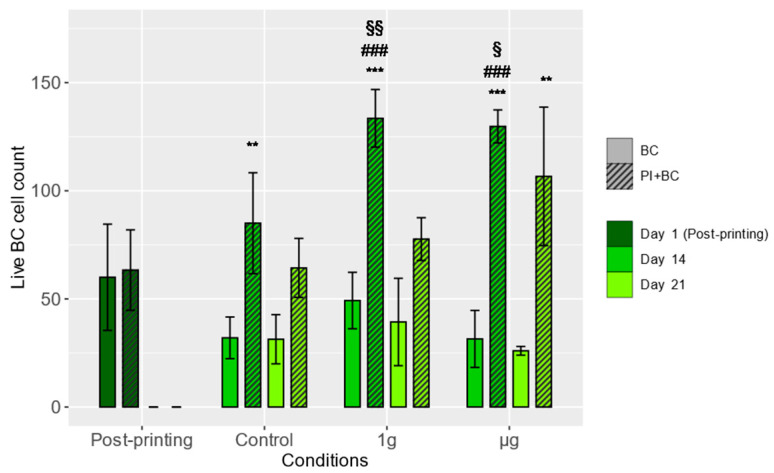
Live cell counts of BCs in 3D-printed scaffolds. Asterisks denote statistically significant (0.05, **—*p* < 0.01, ***—*p* < 0.001) differences between the means measured in BC and PI+BC samples at the indicated days and subjected to different experimental conditions. Means (columns) and standard deviations (error bars) are displayed. Section signs denote statistically significant (§—*p* < 0.05, §§—*p* < 0.01) differences between the specified and equivalent ground control groups. Hashes denote statistically significant (###—*p* < 0.001) differences between the means of the specific measurements and the measurements of Day 1, the post-printing samples. *N* = 3 (post-printing groups), *N* = 4 (other groups).

One week after spaceflight (two weeks after bioprinting), both 1 g and µg condition samples had, on average, statistically significantly more cells when compared to the Day 1 samples, indicating the proliferation of bioprinted BCs. Moreover, the live cell number in BC-alone samples remained relatively stable throughout the experiment, showing no significant proliferation. In all of the tested conditions, one week after spaceflight, as well as the ground control samples, the PI+BC groups had statistically significantly more live BCs compared to the BC-alone samples. This difference remained two weeks after spaceflight in µg samples. One week after spaceflight, both experimental groups of PI+BC samples had statistically significantly greater live cell numbers compared to the ground control samples (Figure 2).

### 3.3. Islet Cell Proliferation in Free-Floating Culture after µg Exposure

**Figure 3:**
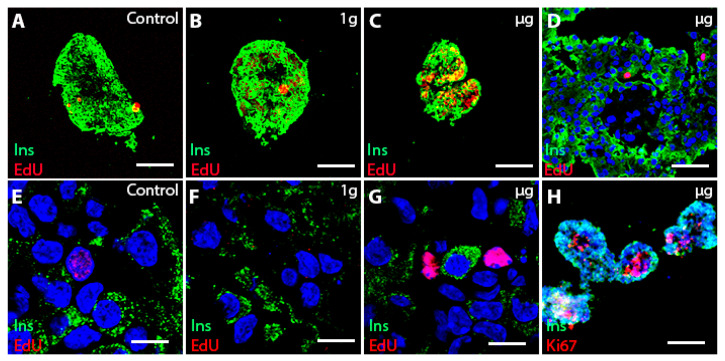
Free-floating islets alone in ground control, 1 g, and µg conditions. Upper panel: insulin + cells (green insulin, red EdU, or Ki67 (**H**) in the non-fused islets (**A**–**C**) proliferate in µg conditions) (**C**). The majority of islets are fused (**D**,**H**). Lower panel: confocal images showing EdU labeling in µg also in insulin-control (**E**), 1 g (**F**) and µg (**G**) groups and insulin + cells (**H**) in µg group. Bar (**A**–**C**) = 100 µm; bar (**D**) = 30 µm; bar (**E**–**G**) = 10 µm; bar (**H**) = 150 µm. Nuclei were stained blue with Hoechst.

**Figure 4:**
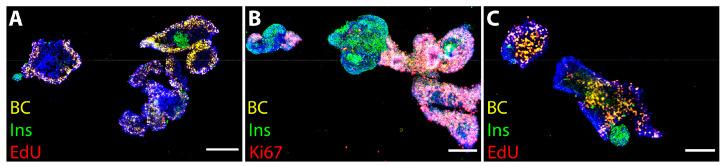
Overview of islets + BCs in 1 g (**A**) and µg (**B**,**C**). Islets+BCs were fused, and islets were surrounded by proliferating BCs (green insulin, yellow BC, red Ki67 (**B**) and EdU (**C**). Yellow-pink BCs are located peripherally and show high proliferation rate. Hoechst + nuclei blue. Bar = 100 µm.

Free-floating islets cultured alone showed good survival after the spaceflight (Figure 3), although the fusion of islets occasionally compromised counts of proliferating cells per islet. Overall, non-fused islets displayed high cell proliferation, specifically in the µg group (Figure 3C, as evidenced by EdU labeling. Islets cultured together with BCs demonstrated good overall survival in all space groups (Figure 4), but extensive fusion between the cultured components (Figure 4A–C) did not permit accurate cell counts per islet.

**Figure 5:**
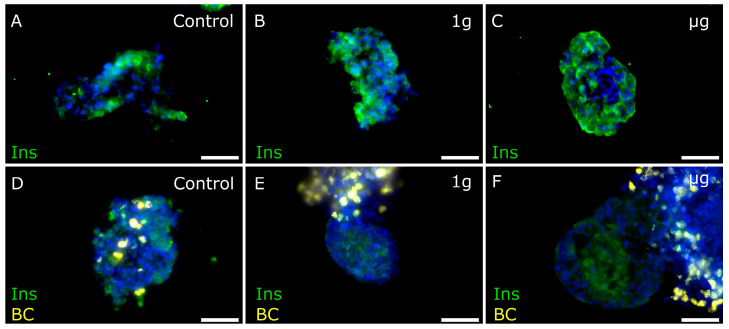
Representative images of insulin staining of free-floating PI and PI+BC samples. (**A**–**C**): PI samples that underwent ground control, 1 g, and µg conditions, respectively, showing insulin staining (green). (**D**–**F**): PI+BC samples that underwent ground control, 1 g, and µg conditions, showing BC presence (yellow) and insulin staining (green). Nuclei stained blue with Hoechst. Bar = 100 µm.

**Figure 6:**
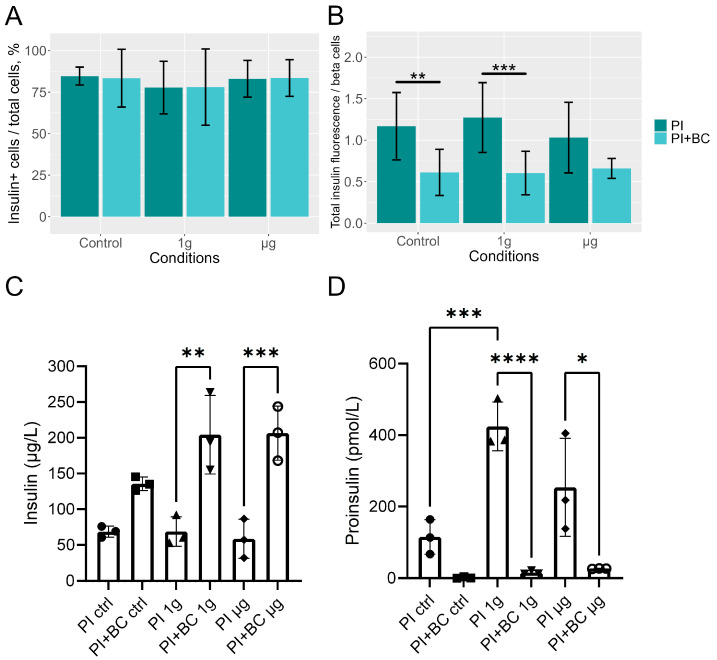
The percentage of free-floating PI cells positive for insulin (**A**) and relative insulin fluorescence (**B**) in the imaged samples. BC signals were removed prior to calculations. Means (columns) and standard deviations (error bars) are displayed in graphs. Stars denote statistically significant (*—*p* < 0.05, **—*p* < 0.01) differences between means. The PI sample sizes were 11, 11, and 9 for control, 1 g, and µg groups, respectively (**A**), and 15, 14, and 13, respectively (**B**). The PI+BC sample sizes were 13, 10, and 11 for control, 1 g, and µg groups, respectively (**A**), and 15, 13 and 13, respectively (**B**). Insulin (**C**) and proinsulin (**D**) content of supernatants as measured by ELISA. Data (bars) are means and standard deviations of three samples per condition analyzed in technical duplicates. One-way ANOVA (*—*p* < 0.05, **—*p* < 0.01, ***—*p* < 0.001, ****—*p* < 0.0001).

Interestingly, islets surrounded by proliferating BCs demonstrated low insulin expression/immunoreactivity compared to islets located in the periphery of the cell conglomerates (Figure 4). To investigate the effect BCs have on insulin production in PIs, more in-depth, the immunocytochemical analysis of the insulin content in the ground control, 1 g and µg samples was performed (Figure 5). (Quantitative measurements thereof are represented in Figure 6A,B.

The immunofluorescence measurements showed that PIs cultured together with or without BCs contained similar percentages of insulin-positive (beta) cells (Figure 6A). However, when the insulin amount per beta-cell count was calculated, we observed that co-culturing PIs with BCs reduces the amount of protein in the insulin-producing cells under control and 1 g, but not µg, conditions (Figure 6B). When instead measuring the contents of insulin and proinsulin in the supernatants from the cells, we found that PIs cultured with BCs had secreted significantly more insulin to the media, possibly explaining the tendency to a lower insulin staining intensity in these (Figure 6C). We also found that PIs cultured without BCs secreted more proinsulin, indicating possible cell stress in these PIs (Figure 6D). Spaceflight, both µg and 1 g, seemed to potentiate these effects.

### 3.4. Cell Proliferation Is Increased in 3D-Printed Cultures Two–Three Weeks after µg Exposure

**Figure 7:**
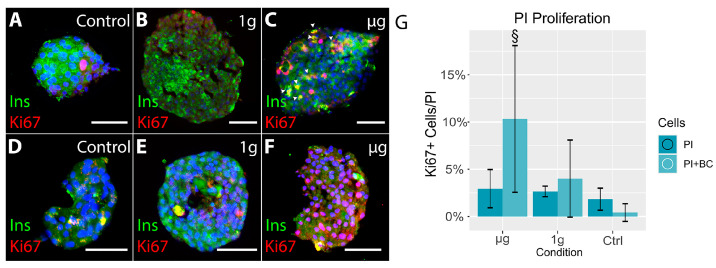
A: Overview of 3D-printed islets alone (upper panel) and PI+BC (lower panel) in control (**A**,**D**), 1 g (**B**,**E**), and µg (**C**,**F**). The proliferation of islet cells was detected three weeks after the space voyage, only in µg-exposed islets (**C**,**F**). Proliferating cells (red Ki67) are insulin-positive (green) in islet-alone cultures (upper panel, right) and insulin-negative in co-culture (lower panel, right). (**G**): Graph shows increased proliferation of islet cells after µg exposure in 3D-printed scaffolds in islets alone and PI+BC co-cultures compared to control groups. Section signs denote statistically significant (§—*p* < 0.05) differences between the specified and equivalent ground control groups. Bar = 100 µm. Nuclei stained blue with Hoechst.

Because of the extensive fusion of PIs in the free-floating specimens, we cryosectioned 3D-printed islets and processed them for immunohistochemistry. We detected extensive proliferation after three weeks in 3D-printed islets cultured alone or with BC, specifically in µg-exposed groups (Figure 7).

## 4. Discussion

Our results show the overall enhanced survival and proliferation of pancreatic beta-cells and increased proliferation of BCs, specifically in µg-exposed groups. There are previous reports of the beneficial effects of microgravity on pancreatic beta-cells. Thus, whereas islet cells derived from patients with persistent hyperinsulinemic hypoglycemia of infancy lose their hormone expression under 1 g culture conditions, they reactivate their insulin expression when grown in simulated µg produced through high-aspect-ratio vessel technology [21]. Islet spheroids generated in simulated µg from mouse insulinoma cells expressed higher levels of several beta-cell signature genes than cells cultured in standard 2D culture conditions [22].

In our experiment, free-floating PI+BC specimens exposed to 1 g and control conditions displayed reduced intracellular insulin levels. A similar outcome was observed in the co-culture of neonatal porcine pancreatic cell clusters with Sertoli cells subjected to simulated µg compared to cell clusters cultured alone [23]. These findings are also in line with previous observations that during embryogenesis, the presence of neural crest stem cells in juxtaposition to maturing pancreatic cells affects their secretion and production of insulin [5]. However, insulin levels in the supernatants showed that the decreased insulin content in PIs cultured with BCs might indicate increased insulin release. This did, however, not seem to be a sign of beta-cell stress, as these conditions did not show the same high levels of released proinsulin. The release of unprocessed proinsulin may be an indication of beta-cell stress, as shown previously [24]. Importantly, under µg, the amount of intracellular insulin in beta-cells was comparable in both mono- and co-cultures. The concentration of secreted insulin was significantly higher in PI+BC (compared to PI cultures) affected by µg and 1 g, but not control conditions. Taken together, the results suggest that co-culturing PIs with BCs is the most beneficial for insulin maturation and secretion under µg. However, to prove that the µg conditions favor beta-cell viability and functionality, future experiments with stimulated glucose challenge after space exposure are necessary.

Although PIs exposed to µg survived and were able to proliferate, the analysis of their morphological features was hampered by extensive islet fusions. In 3D-printed scaffolds, islet morphology and proliferation capacity were maintained, demonstrating the advantages of this preparation for microgravity-mediated engineering of functional islet tissue. Our findings, together with the results of other studies that investigated the bioprinting of pancreatic cells, strongly suggest that bioprinting helps maintain the viability and function of these cells and preserves their structure, showing clear advantages to free-floating or 2D culture [25].

Utilizing the highly accelerating, rotating-vessel biochamber model for simulated µg, lipopolysaccharide (LPS)-stimulated rodent islets showed reduced production of tumor necrosis factor-alpha and a reduced insulin concentration, but an increased concentration of glucose and amino acid turnover compared to control cultures [26]. Subclinical diabetogenic changes have been observed in humans after spaceflight, possibly related to changes in cytokine expression [27], which may reflect the activation of immune mediators rather than direct µg-mediated effects on islet cells. At the cellular level, however, studies in simulated µg have shown beneficial effects on survival and insulin secretion of beta-cells in vitro [23,28,29] and in vivo [30,31]. Our analysis provides additional evidence for the ability of islet cells, similar to our previous findings with BC, to not only withstand µg but also expose a delayed capacity for cell renewal.

In the case of BC, the greatest increase in live cell count—indicating intensive proliferation—was observed one week after spaceflight, specifically in PI+BC co-cultures, which not only had significantly more live cells relative to the amount measured immediately after bioprinting, but significantly more than the ground control samples. Studies on human neural stem cells subjected to simulated µg indicate that increased proliferative activity may be induced by upregulating several mitochondrial pathways [32]. Interestingly, parabolic flights seemed to decrease the proliferative potential of mesenchymal stem cells, suggesting a difference in stress exposure compared to actual spaceflights aboard sounding rockets [33].

Analyzing the bioprinted co-cultures, we detected increased proliferation of islet cells two weeks after space exposure in the µg group (three weeks after 3D bioprinting), suggesting that islet cells may enter the cell cycle after a delay after the space exposure.

The live BC number declined and returned to Day 1 levels three weeks after bioprinting (two weeks after spaceflight) for all experimental groups. It has been observed that bioprinted cell cultures may display decreased proliferation as late as nine days after printing, presumably due to contact pressure from the increasing density of cells. The detected drop in BC number is, therefore, not unexpected [34]. We observed a significant decrease in BC viability three weeks after bioprinting in BC-alone and PI+BC samples that had been subject to 1 g conditions, indicating increasing cell death, although their proliferation rate may not have been further affected. No such drop in viability was observed in the µg experimental group samples. These findings align with our previous findings, showing that even a short exposure to µg can induce long-lasting cellular effects and manifest themselves after a delay of one or several weeks [35].

Importantly, when BCs were bioprinted alone, under all the tested conditions and time points, we did not observe a significant increase in cell number, and the cells had suboptimal viability, indicating that the BC cultures lack the stimulus to remodel their environment and proliferate. It has been shown that bioprinted neural crest stem cells require a high cell density to proliferate effectively [36]. In our study, only in the samples where both BC and PI cells were present—meaning higher cell density—was an increase in BC number detected, which was in line with these findings. Moreover, the direct contact between the cells as well as the cytokines (such as IGF-II) and extracellular molecules (such as NCAMs) that pancreatic cells provide could have a positive impact on BC viability and proliferation [37].

During our experiment, rocket spin occurred during the rocket boost phase until 57 s after lift-off. The spin slowly built up to 980 deg/s and then, in one step, was reduced to 7.2 deg/s at 57 s, and further reduced to 0 deg/s at 65 s after lift-off. However, since the spin-up and spin-down occur before the experimental phase, any side effects induced by shearing and centrifugal forces during the spinning phase are unlikely. It should be noted that the µg levels on sounding rockets are generally lower than 0.01 mg. Such low levels are not achieved during parabolic flights but are achievable at a space station.

Our new findings may have implications for designing protocols for biological analyses after sounding rocket experiments, which aim to detect the effects of short exposure to µg as soon as possible after space exposure or even onboard. The results of this study suggest that bioprinting of PIs can both improve their survivability and facilitate the processing of samples for analysis. Further studies in ground-based simulated µg experiments may help to elucidate the mechanisms of delayed µg effects, improve our understanding of the impact of µg on neural stem cells and beta-cells, and contribute to potential clinical applications.

## 5. Conclusions

Pancreatic islets survive the challenge of space conditions in free-floating culture and are even better in 3D-printed scaffolds. They can thus be further explored to investigate the effects of real and simulated µg on their survival, proliferation, and function. Importantly, our study shows these effects may not be present immediately after µg exposure but only after a post-flight delay. We detected beneficial changes to insulin maturation and secretion in PI samples cultured together with BCs that were exposed to µg. These findings should be taken into account in analyses of biological samples exposed to µg. Furthermore, elucidating the mechanisms underlying these observations may help develop approaches to protect beta-cell functionality during long-term space missions and promote beta-cell survival and renewal in type 1 diabetes.

## Data Availability

Data are contained within the article.

## Supplementary Materials

The following supporting information can be downloaded at: https://www.mdpi.com/article/10.3390/cells13181588/s1, Figure S1. Cassettes Layout of the hardware provided by SIOUX Technologies (https://www.siouxtechnologies.com, accessed on 18 September 2024) for the MASER15 campaign comprising a hard plastic base block with space for two triplet membranes with each one main compartment (3 mL) and two side compartment (1 mL) and respective membrane seals, extra black membrane containers for fixatives and respective lids, a metal bottom plates, screws, and an electronically triggered plunger mechanism plate for injection of the side compartments contents to the main compartments. Figure S2. Schematic of an assembled Cassette provided by the company SIOUX Technologies (https://www.siouxtechnologies.com, accessed on 18 September 2024). The center compartments are meant for cell suspensions and side compartments for stainings and fixatives. The membranes are emedded in the base block. The gray and green top plate represent the electronic activation mechanism with the anchored plungers in a darker shade of brown. Figure S3. Image of an assembled Cassette with a black plastic dummy instead of the electronic plunger mechanism plate on top of the cassette for practice routines. All membrane compartments were labeled on the building block for facilitating easier filling; A&D are filled with staining solutions, B&E are the main compartments filled with the cell suspensions; in C&E the black extra membranes filled with the fixatives were inserted. Each cassette received a tag for identification and tracking of pre-flight safety checks conducted by SIOUX Technologies and SSC.
