# Supplementary material for: Microgravity Effect on Pancreatic Islets
Source: Cells. 2024 Sep 21;13(18):1588. doi: 10.3390/cells13181588 (PMC11430520; doi:10.3390/cells13181588)
Supplement: Supplementary file 1 [file cells-13-01588-s001.zip › cells-3146432-supplementary.pdf]

Supplementary Material

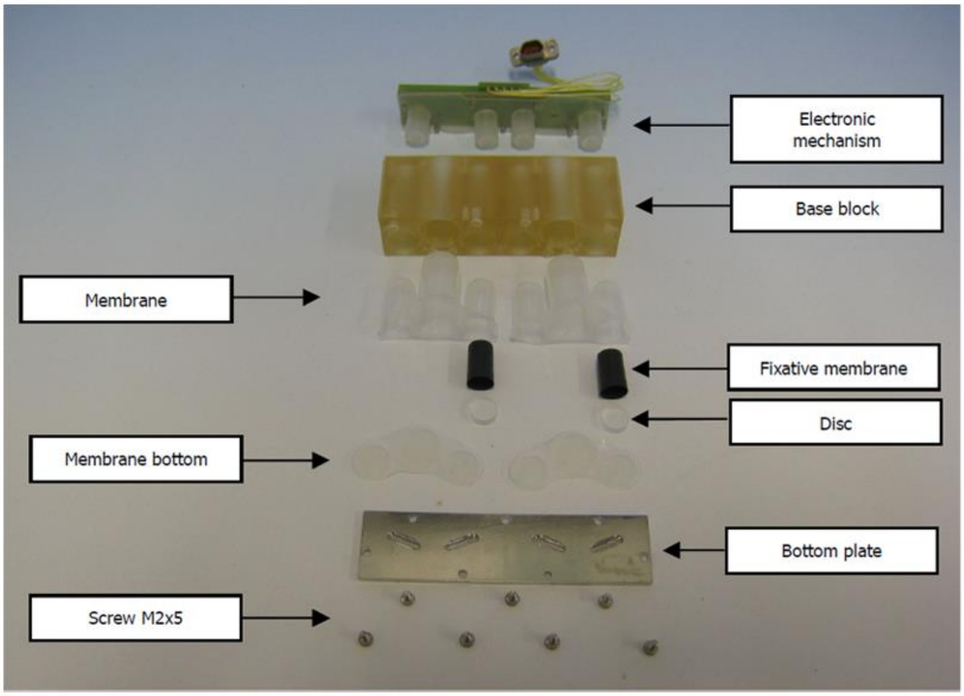

Figure S1: Cassettes Layout of the hardware provided by SIOUX Technologies (https://www.siouxtechnologies.com) for the MASER15 campaign comprising a hard plastic base block with space for two triplet membranes with each one main compartment (3ml) and two side compartment (1ml) and respective membrane seals, extra black membrane containers for fixatives and respective lids, a metal bottom plates, screws, and an electronically triggered plunger mechanism plate for injection of the side compartments contents to the main compartments.

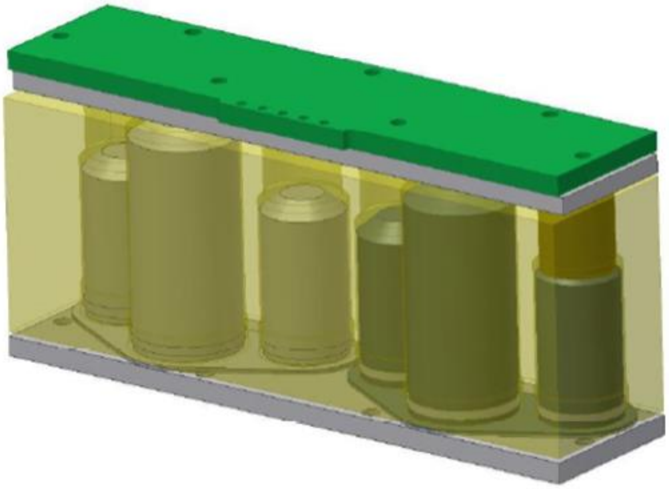

Figure S2: Schematic of an assembled Cassette provided by the company SIOUX Technologies (https://www.siouxtechnologies.com). The center compartments are meant for cell suspensions and side compartments for stainings and fixatives. The membranes are emedded in the base block. The gray and green top plate represent the electronic activation mechanism with the anchored plungers in a darker shade of brown.

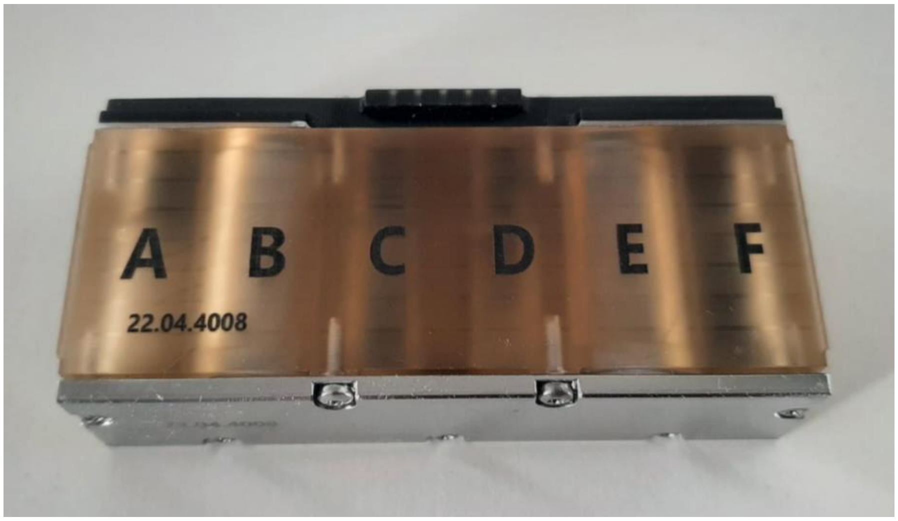

Figure S3: Image of an assembled Cassette with a black plastic dummy instead of the electronic plunger mechanism plate on top of the cassette for practice routines. All membrane compartments were labeled on the building block for facilitating easier filling; A&D are filled with staining solutions, B&E are the main compartments filled with the cell suspensions; in C&E the black extra membranes filled with the fixatives were inserted. Each cassette received a tag for identification and tracking of pre-flight safety checks conducted by SIOUX Technologies and SSC.
